# Alcoholic Stimulation

**Published:** 1896-08-22

**Authors:** 


### EDITOR'S LETTER-BOX.

[Our correspondents are reminded that proxility is a great bar to publication, and that brevity of style and conciseness of statement greatly facilitate early insertion.]

### ALCOHOLIC STIMULATION.

Sir,—I have just read Dr. Richardson's article on alcoholic stimulation, and a case occurs to me which seems to show that the use of alcohol is, under some circumstances, advantageous, and, in fact, preserves life when nothing else will. I can vouch for the correctness of the following: A man in one of our large towns was extremely ill, and was attended by one of the leading doctors—a man of wide experience and great reputation—who gave no hope of his recovery. The patient was suddenly taken worse, and the doctor was summoned, but being absent, another doctor—who happened to be son-in-law to the first-named—was summoned, and after examining the patient asked for some brandy. "Oh, sir," said the wife, "Dr. A. said he must not have any on any account." "Never mind Dr. A.," said Dr. B., "give me some brandy," and he proceeded to administer it by teaspoonful doses every ten minutes or so. Whilst he was thus engaged, Dr. A. came in. "What are you doing?" said he. "Giving him brandy," replied Dr. B. "You'll kill him," said Dr. A. "It's the only thing that will save his life," replied Dr. B. Dr. A.: "Do as you like, do as you like; he'll die, anyway," and he left the house. Dr. B. remained with his patient all night, and continued administering brandy at intervals, with the result that the patient ultimately recovered. This is not the only instance—though it is a striking one—in which I have known life to be saved (apparently) by the administration of alcohol, and it would take a very large amount of theory, or scientific demonstration, to counteract facts like these which are within one's personal knowledge. I enclose my card, and subscribe myself—Yours truly, Experientia Docet.

*** As an opponent of the use of alcohol Sir Benjamin's obvious answer to our correspondent would be summed up in the word he has so wisely used, "apparently," but there are many members of the profession, of high position and large experience, who would entirely endorse the letter of our correspondent.—Ed. *T. H.*

